# Investigation of the Mechanisms of Tramadol-Induced Seizures in Overdose in the Rat

**DOI:** 10.3390/ph15101254

**Published:** 2022-10-12

**Authors:** Camille Lagard, Dominique Vodovar, Lucie Chevillard, Jacques Callebert, Fabien Caillé, Géraldine Pottier, Hao Liang, Patricia Risède, Nicolas Tournier, Bruno Mégarbane

**Affiliations:** 1 Inserm, UMRS-1144, Optimisation Thérapeutique en Neuropsychopharmacologie, Université Paris Cité, F-75006 Paris, France; 2 Department of Medical and Toxicological Critical Care, AP-HP, Lariboisière Hospital, 75010 Paris, France; 3 Imagerie Moléculaire In Vivo, IMIV, CEA, INSERM, CNRS, Universités Paris-Sud et Paris-Saclay, 91471 Orsay, France; 4 Laboratory of Biochemistry and Molecular Biology, AP-HP, Lariboisière Hospital, 75010 Paris, France

**Keywords:** GABA, overdose, positron emission tomography, seizure, tramadol

## Abstract

Tramadol overdose is frequently associated with the onset of seizures, usually considered as serotonin syndrome manifestations. Recently, the serotoninergic mechanism of tramadol-attributed seizures has been questioned. This study’s aim was to identify the mechanisms involved in tramadol-induced seizures in overdose in rats. The investigations included (1) the effects of specific pretreatments on tramadol-induced seizure onset and brain monoamine concentrations, (2) the interaction between tramadol and γ-aminobutyric acid (GABA)_A_ receptors in vivo in the brain using positron emission tomography (PET) imaging and ^11^C-flumazenil. Diazepam abolished tramadol-induced seizures, in contrast to naloxone, cyproheptadine and fexofenadine pretreatments. Despite seizure abolishment, diazepam significantly enhanced tramadol-induced increase in the brain serotonin (*p* < 0.01), histamine (*p* < 0.01), dopamine (*p* < 0.05) and norepinephrine (*p* < 0.05). No displacement of ^11^C-flumazenil brain kinetics was observed following tramadol administration in contrast to diazepam, suggesting that the observed interaction was not related to a competitive mechanism between tramadol and flumazenil at the benzodiazepine-binding site. Our findings do not support the involvement of serotoninergic, histaminergic, dopaminergic, norepinephrine or opioidergic pathways in tramadol-induced seizures in overdose, but they strongly suggest a tramadol-induced allosteric change of the benzodiazepine-binding site of GABA_A_ receptors. Management of tramadol-poisoned patients should take into account that tramadol-induced seizures are mainly related to a GABAergic pathway.

## 1. Introduction

Opioid overdose is the first cause of drug-induced poisonings and fatalities in the US [1]. A substantial part of this increase has been attributed to tramadol, a WHO step-2 opioid analgesics for which prescriptions have extensively grown following dextropropoxyphene withdrawal from the market [2]. Tramadol-induced analgesic activity is related to its mu-opioid receptor (MOR) agonist properties and ability to inhibit norepinephrine and serotonin reuptake [3].

Tramadol poisoning causes coma (~30%), seizures (~15%), agitation (~10%) and respiratory depression (~5%) [4,5]. Based on a large cohort of 8566 tramadol mono-exposures reported in 2009–2011 to the National Poison Data System of the American Association of Poison Control Centers, an ~15% incidence of tramadol-induced seizures was determined, with a relative risk of 7.94 (95%-confidence interval, 2.99–20.91) in comparison to tapentadol another antalgic providing pain relief through similar monoaminergic and opioid agonist properties [6]. Tramadol overdose typically results in isolated, brief, self-limiting generalized tonic-clonic seizures, often occurring within 4–6 h post-ingestion, although electroencephalogram abnormalities and recurrent seizures or status epilepticus are not rare [5,7,8,9]. Based on the Toxicology Investigators Consortium data registry, tramadol-induced seizures were more likely observed in Asian patients and patients abusing or misusing the medication [10]. A recent systematic review showed that the occurrence of seizures in patients exposed to tramadol is dose-dependent and related to male gender but not to naloxone administration used to reverse coma and respiratory depression [11]. Serotonin syndrome (SS) may occur, but its incidence is controversial [12,13].

Mechanisms of tramadol-induced seizures remain poorly understood. Seizures are usually included in the SS and related to serotonin receptor overstimulation, mainly the 1A and 2A receptor subtypes, by increasing synaptic serotonin concentration [14]. Based on animal data, the serotoninergic mechanism of tramadol-induced seizures has been questioned [15,16]. Rat pretreatment with cyproheptadine, a serotonin receptor antagonist, did not abolish tramadol-induced seizures [16] while 5-hydroxytryptophan/benserazide, a combination enhancing brain serotonin, was protective [15] or without effects [16]. The involvement of non-serotoninergic pathways, including γ-aminobutyric acid- (GABA)ergic [17], histaminergic [18], dopaminergic [19] and opioidergic [17,18] have been hypothesized to account for tramadol-induced seizures.

A rat study was designed to clarify the mechanism of tramadol-induced seizures. Various pretreatments were used to investigate the impact of the hypothesized neurotransmission systems on the onset of tramadol-induced seizures and the brain monoamine content.

## 2. Results

### 2.1. Study 1-Effects of Pretreatments on Tramadol-Induced Sedation, Seizures, Effects on Temperature and Brain Monoamines

**Figure 1:**
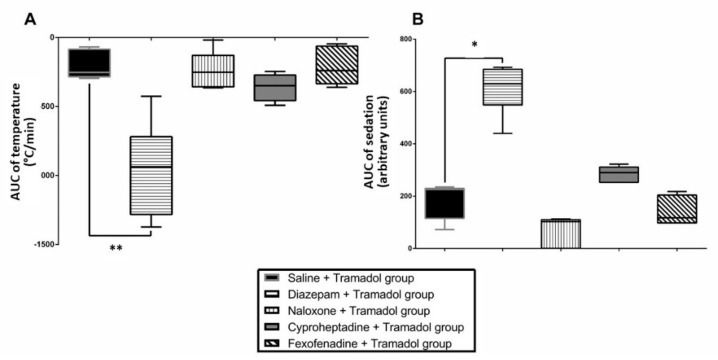
Effects on sedation (**A**) and temperature (**B**) of intraperitoneal (i.p.) 75 mg/kg tramadol administration (T0) in Sprague-Dawley rats pretreated with i.p. 4% tween at T-15 + intravenous (i.v.) saline at T-10 (Saline + Tramadol group, black); i.p. 1.77 mg/kg diazepam at T-15 + i.v. saline at T-10 (Diazepam + Tramadol group, horizontal lines); i.p. 4% tween at T-15 + i.v. 2 mg/kg bolus followed by 4 mg/kg/h naloxone at T-10 (Naloxone + Tramadol group, vertical lines); i.p. 10 mg/kg cyproheptadine at T-15 + i.v. saline at T-10 (Cyproheptadine + Tramadol group, grey); and i.p. 15 mg/kg fexofenadine at T-30 + i.v. saline at T-10 (Fexofenadine + Tramadol group, diagonal lines). Results are expressed as median and quartiles (N = 6/group). * *p* < 0.05, ** *p* < 0.01.

Diazepam significantly deepened sedation (*p* < 0.05) and decreased temperature (*p* < 0.01) in tramadol-treated rats (Figure 1). Naloxone, cyproheptadine and fexofenadine did not alter tramadol-induced effects on sedation and temperature.

**Figure 2:**
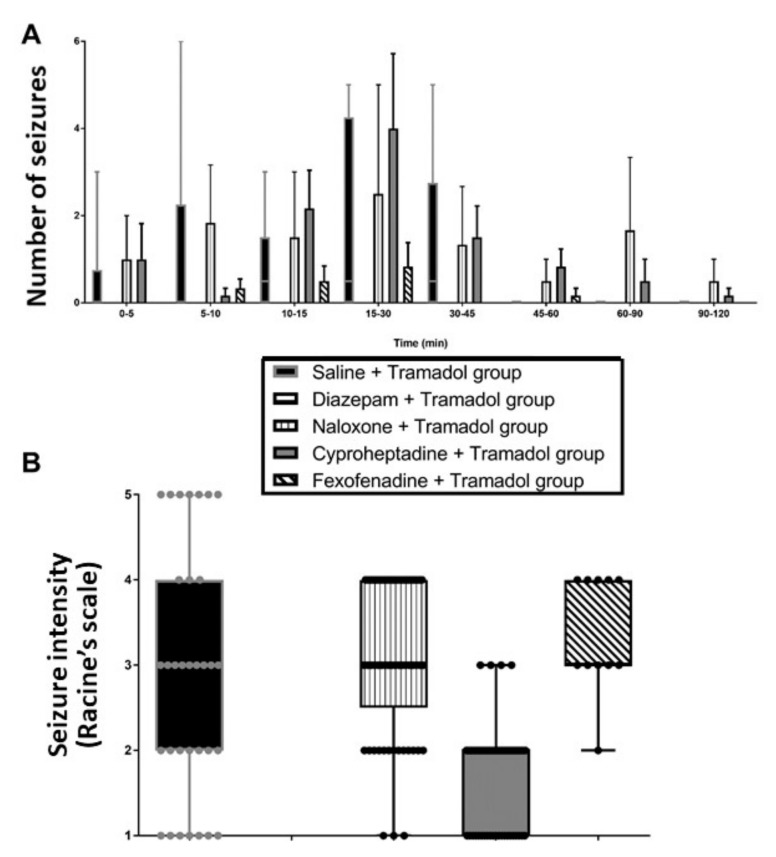
Effects on the seizure time-course (**A**) and intensity ((**B**); each dot representing one seizure episode) of intraperitoneal (i.p.) 75 mg/kg tramadol administration in Sprague-Dawley rats pretreated with i.p. 4% tween at T-15 + intravenous (i.v.) saline at T-10 (Saline + Tramadol group, black); i.p. 1.77 mg/kg diazepam at T-15 + i.v. saline at T-10 (Diazepam + Tramadol group, horizontal lines); i.p. 4% tween at T-15 + i.v. 2 mg/kg bolus followed by 4 mg/kg/h naloxone at T-10 (Naloxone + Tramadol group, vertical lines); i.p. 10 mg/kg cyproheptadine at T-15 + i.v. saline at T-10 (Cyproheptadine + Tramadol group, grey); and i.p. 15 mg/kg fexofenadine at T-30 + i.v. saline at T-10 (Fexofenadine + Tramadol group, diagonal lines). Results are expressed as median and quartiles. (N = 6/group).

Diazepam completely abolished seizures in tramadol-treated rats, whereas naloxone, cyproheptadine and fexofenadine pretreatments had no significant effects on seizures. Seizure occurrence was prolonged up to 120 *versus* 45 min by cyproheptadine and fexofenadine pretreatments (Figure 2).

**Figure 3:**
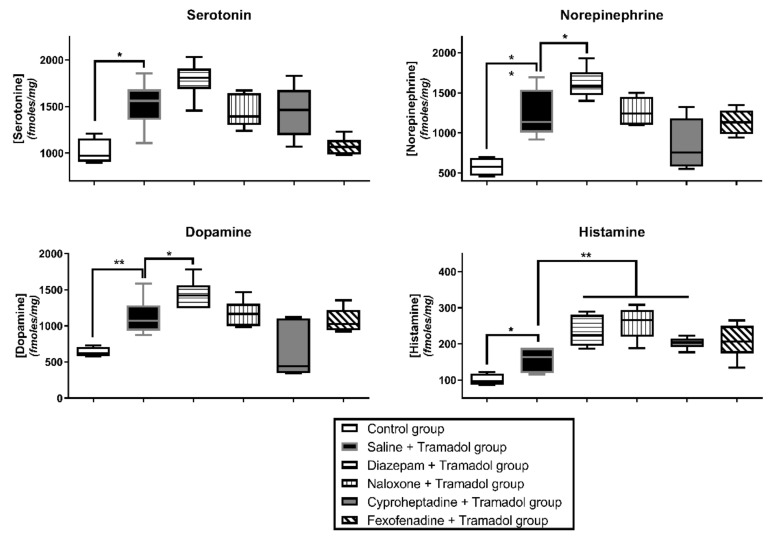
Effects on the frontal cortex monoamine concentrations of intraperitoneal (i.p.) 75 mg/kg tramadol administration in Sprague-Dawley rats pretreated with i.p. 4% tween at T-15 + intravenous (i.v.) saline at T-10 (Saline + Tramadol group, black); i.p. 1.77 mg/kg diazepam at T-15 + i.v. saline at T-10 (Diazepam + Tramadol group, horizontal lines); i.p. 4% tween at T-15 + i.v. 2 mg/kg bolus followed by 4 mg/kg/h naloxone at T-10 (Naloxone + Tramadol group, vertical lines); i.p. 10 mg/kg cyproheptadine at T-15 + i.v. saline at T-10 (Cyproheptadine + Tramadol group, grey); and i.p. 15 mg/kg fexofenadine at T-30 + i.v. saline at T-10 (Fexofenadine + Tramadol group, diagonal lines). Control rats received i.p. saline at T0 + i.p. 4% tween at T-15 + i.v. saline at T-10 (Control group, white). Results are expressed as median and quartiles. (N = 6/group). * *p* < 0.05, ** *p* < 0.01.

Tramadol overdose was responsible for a significant increase in histamine, serotonin, norepinephrine and dopamine concentrations (*p* < 0.05, *p* < 0.05, *p* < 0.01 and *p* < 0.01, respectively; Figure 3) and decreases in 5-HIAA, HVA and DOPAC concentrations (*p* < 0.01) in comparison with the control. Tryptophan concentration was not significantly modified. Diazepam pretreatment significantly increased tramadol-induced effects on histamine, norepinephrine and dopamine concentrations (*p* < 0.01, *p* < 0.05 and *p* < 0.05, respectively) as well as its effects on 5-HIAA, MHPG and HVA concentrations (*p* < 0.01, *p* < 0.05 and *p* < 0.01, respectively). Naloxone significantly increased tramadol-induced effects on histamine concentrations (*p* < 0.01) as well as its effects on MHPG and DOPAC concentrations (*p* < 0.05). Cyproheptadine significantly increased tramadol-induced effects on histamine concentration (*p* < 0.01). Fexofenadine significantly increased tramadol-induced effects on HVA and DOPAC concentrations (*p* < 0.01 and *p* < 0.05, respectively). No pretreatment resulted in significant changes in the absence of tramadol-induced effects on tryptophan concentrations.

### 2.2. Study 2-Interactions of Tramadol with GABA_A_ Receptors

**Figure 4:**
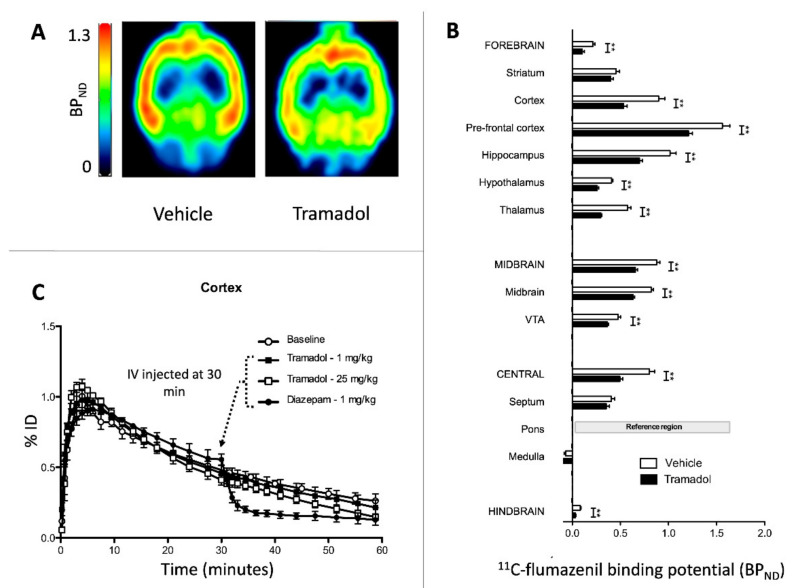
Effects of tramadol on ^11^C-flumazenil binding to the brain. Parametric PET images displaying the regional binding of ^11^C-flumazenil (BP_ND_) without (Vehicle) or with (Tramadol) pre-treatment with intraperitoneal 75 mg/kg tramadol, 15 min before intravenous (i.v.) ^11^C-flumazenil are shown in (**A**). The corresponding BP_ND_ in selected brain regions in rats receiving either baseline ^11^C-flumazenil (Vehicle group, white) or tramadol injection (Tramadol group, black) are shown in (**B**). Results are expressed as mean ± SEM. (N = 5/group). ** *p* < 0.01. Displacement experiments are reported in (**C**), displaying ^11^C-flumazenil time activity curves in the cortex, i.v. injected alone (white circle), with 1 mg/kg tramadol i.v. (black square), 25 mg/kg tramadol i.v. (white square) or 1 mg/kg diazepam i.v. (black circle) injected 30 min after the beginning of the PET acquisition. Results are expressed as mean ± SEM (N = 2/group).

In the control rats (Vehicle group), ^11^C-flumazenil PET images as well as the corresponding *BP_ND_* values were consistent with typical data obtained in similar conditions [20]. In rats receiving 75 mg/kg tramadol i.p. before ^11^C-flumazenil, significant reductions in *BP_ND_* were observed in most brain regions, except in the striatum, medulla and septum (Figure 4A,B).

No displacement of ^11^C-flumazenil brain kinetics was observed following i.v. 1 or 25 mg/kg tramadol injection after 30 min PET acquisition. In comparison, a marked displacement was observed after the injection of the reference ligand diazepam (1 mg/kg) administered in the same conditions (Figure 4C).

## 3. Discussion

Our findings provide strong evidence against serotonin-, histamine-, norepinephrine- and dopamine-mediated pathways of tramadol-induced seizures in overdose. An interaction between tramadol and the benzodiazepine-binding site of the GABA_A_ receptor has been clearly highlighted, suggesting a role for the GABAergic pathway in mediating tramadol-induced seizures.

Our rat model reliably reproduced tramadol toxicity observed in humans, including sedation, hypothermia and seizures [4,5,10]. As previously published [16], our tramadol-overdosed rats exhibited early-onset seizures occurring as soon as 5 min after tramadol injection and disappearing 45 min later. Tramadol overdose induced significant increases in serotonin, histamine, norepinephrine and dopamine concentrations in the brain (Figure 3) with significant decreases in 5-HIAA (serotonin catabolite), MHPG (norepinephrine catabolite), HVA and DOPAC (dopamine catabolites) concentrations. These findings suggested a reduction in the catabolism of these monoamines, as previously attributed to the inhibition of the oxygen-dependent monoamine oxidase-A due to brain tissue hypoxia in tramadol-overdosed rats [16].

Concomitant use of tramadol with tricyclic or serotonin reuptake inhibitor antidepressants has been associated with enhanced risk of seizures, leading to the assumption that tramadol-induced seizures may represent one of the SS symptoms [14], despite the lack of convincing evidence establishing the role of serotonin in the generation of tramadol-induced seizures. Our findings provide evidence against the involvement of serotoninergic pathways in tramadol-induced seizures, as previously suggested [15,16]. Usually, SS-related seizures are prevented by cyproheptadine, a serotonin receptor antagonist, as shown in citalopram overdose [21]. In our study, cyproheptadine did not alter tramadol-induced effects neither on sedation, nor on hypothermia (Figure 1) or seizure onset (Figure 2). Cyproheptadine did not significantly alter most of tramadol-induced effects on brain monoamines in the frontal cortex (Figure 3). Only histamine concentrations were significantly increased possibly related to its antagonist properties on H1-receptors.

Involvement of the dopaminergic system in tramadol-induced seizures was suggested by the increase in tramadol-related seizure threshold obtained in mice with haloperidol, a predominantly D2-receptor antagonist [19]. In addition to obvious limitations due to haloperidol-related direct epileptic activity in this study, the significant increase in brain dopamine observed in diazepam-pretreated rats while exhibiting seizure abolishment precludes the contribution of dopaminergic pathways to seizures.

Multiple mechanisms resulting in opioid receptor overstimulation seem involved in the precipitation of seizure activity in laboratory animals [22]. Involvement of kappa-opioid receptors in tramadol-induced seizures was suggested [23], even though tramadol affinity for this receptor subclass is low [3,24]. Here, naloxone did not alter tramadol-induced effects on sedation, temperature (Figure 1) and seizures but prolonged their occurrence time (Figure 2). Experimental data regarding naloxone-related seizurogenic effects remain controversial, depending on the animal model and experimental conditions. Most studies demonstrated rather protective effects against both opioid- and non-opioid-related seizures [17,25,26]. Other studies reported no protective and even worsening effects of naloxone on seizures [27,28], supporting our findings. Naloxone significantly enhanced tramadol-induced effects on the brain histamine, providing evidence against the hypothesis of an H1-receptor activation-linked pathway mediated by opioid receptor-dependent histamine release from mast cells [18].

Usually, H1-receptor agonists or histamine releasers exhibit anticonvulsant effects [18,29,30], while H1-receptor antagonists are responsible for seizurogenic effects [31,32,33]. Despite the absence of direct evidence, connections between tramadol-induced seizures and histamine have been hypothesized based on data showing that antihistamines and histamine release inhibitors attenuate this adverse effect [18]. Our findings provide evidence against the involvement of histaminergic pathways in tramadol-induced seizures. Pretreatment with fexofenadine did not alter tramadol-induced effects on sedation, temperature and seizures but prolonged their occurrence time (Figure 1 and Figure 2). The contribution of fexofenadine to the observed decrease in the brain HVA and DOPAC concentrations remained unclear.

Multiple little-convincing other paths were hypothesized as contributing to tramadol-induced seizures. A recent study suggested that the adenosinergic system, which plays a recognized role in seizure susceptibility, might be involved in tramadol-induced seizure inhibition [34]. Administration of caffeine, a nonselective antagonist of adenosine receptors (mainly A1 and A2 subtypes) decreased tramadol-related seizure threshold in Albino mice and limited oxidative damage in brain mitochondria. This path could be questioned due to the multiple pharmacological activities of caffeine, including a competitive antagonistic action on the ionotropic glycine receptor, a widely distributed inhibitory receptor in the central nervous system.

The GABAergic hypothesis, the most reliable one, was investigated. Benzodiazepines, allosteric GABA_A_ receptor agonists, are able to prevent and treat tramadol-induced seizures [4,35,36]. Consistently, our previous experimental data combining elevated doses of both diazepam and tramadol clearly showed the diazepam-induced abolishment of tramadol-related seizures, even though this combination enhanced respiratory depression [18]. In our rat model, diazepam at a pharmacological dose efficiently prevented seizures (Figure 2) while enhancing sedation and hypothermia (Figure 1). Diazepam pretreatment was responsible for significant increase in tramadol-induced effects on norepinephrine and dopamine, with a trend towards tramadol-induced increase in serotonin concentration and significant increase in tramadol-induced effects on 5-HIAA, MHPG and HVA (Figure 3). These effects could be explained by the enhanced respiratory depression related to diazepam/tramadol combination and its resulting decrease in brain oxygenation explaining monoamine catabolism inhibition [16]. Despite diazepam-related seizure prevention, no monoamine concentration went back to its baseline. The increased concentrations of monoamines, including norepinephrine and the decreased concentrations of their catabolites, strongly suggested that they were not involved in tramadol-induced seizures. These observations were consistent with the increased risk of seizures in cyproheptadine-treated rats, which exhibited decreased brain norepinephrine content. Multiple experimental data in the literature support the regulatory and protective role of norepinephrine against seizures, with enhanced neuronal damage following focal status epilepticus if norepinephrine content is reduced in the brain [37,38,39]. Norepinephrine exerts its anticonvulsant effects through β2-adrenergic receptors by stimulating the autophagy pathway. Noradrenergic fibers originating from the locus coeruleus densely innervate limbic structures, including the piriform cortex, which represents the limbic structure with the lowest seizure threshold [40].

GABA is the main inhibitory neurotransmitter, present in ~30% of central synapses. The inhibition of GABAergic pathways was hypothesized to actively participate in tramadol-induced seizures. Based on an in vitro study on human recombinant neurotransmitter-gated ion channels, GABA_A_ receptors were shown to be inhibited by tramadol and its M1 metabolite (O-desmethyl-tramadol) but only at high concentrations (100 µM), possibly correlating with convulsion onset in vivo [41]. Here, i.p. 75 mg/kg tramadol induced a significant decrease in ^11^C-flumazenil binding to most brain regions, suggesting a molecular interaction between tramadol and GABA_A_ receptors in the brain accounting for its seizing activity.

Several hypotheses may explain the decrease in *BP_ND_* (=B_max_/K_D_) observed after tramadol administration. Increasing doses of tramadol were not able to displace ^11^C-flumazenil binding from the brain (Figure 4C), supporting the absence of direct competition between tramadol and flumazenil at the GABA_A_ receptors. Of note, flumazenil was reported to exhibit a pharmacological profile of a competitive antagonist with a weak intrinsic agonist activity, suggesting more complex interactions with GABA_A_ receptors than initially thought and explaining some variability in emergence characteristics when administered in humans [42]. A decrease in ^11^C-flumazenil *BP_ND_* has been previously observed in several rat models of epilepsy and explained by the decrease in B_max_ while K_D_ remained constant in comparison to the control [20,43,44]. These findings were consistent with the internalization of GABA_A_ receptors in the in vitro studies observed at least one hour after status epilepticus [45,46]. In our PET study, no seizure was observed following i.p. 75 mg/kg tramadol administration. In non-anesthetized rats, the same dose induced seizures occurring as early as 5 min after tramadol injection [16]. Isoflurane anesthesia, known to exhibit anti-convulsive properties [47,48], can explain these discrepancies, although occurrence of subclinical seizures cannot be ruled out in our anesthetized rats during the procedure since electroencephalographic recording was impossible during PET acquisition. This nonetheless suggests that the decrease in *BP_ND_* resulted from specific tramadol action on the GABA_A_ receptors rather than non-specific GABA_A_ receptor alteration during seizures. This hypothesis is supported by in vitro data reporting GABA_A_ receptor inhibition mediated ion channeling by high concentration tramadol and its M1 metabolite (100µM) [41].

The ionotropic GABA_A_ receptor is a ligand-gated ion channel, composed of five subunits forming the Cl^-^ channel [49]. Both acute and chronic tramadol administration markedly potentiated seizing activity induced in rodents by pentylenetetrazole, a noncompetitive antagonist that blocks GABA-mediated Cl^-^ influx by allosteric interaction at the Cl^-^ channel [17,50]. Allosteric modulation of GABA_A_ receptors by high dose tramadol may be hypothesized to explain the decrease in *BP_ND_*. This hypothesis is consistent with the rapid onset of seizures, observed as soon as 5 min after tramadol injection [16]. The multiplicity of drug binding sites on the GABA_A_ receptor supports such an interaction consistent with several proconvulsant antagonists that interact on various sites of the receptor [51], like bicuculline competing with GABA on its binding site and picrotoxin acting on the “picrotoxin binding site” and blocking the Cl^-^ channel [49]. Our findings suggest that tramadol at elevated concentrations may bind to the GABA_A_ receptor at a different binding site from the benzodiazepine site and that allosteric modulation resulting from such an interaction explains seizure onset.

The hypothesis of tramadol/GABA_A_ receptor interaction may be insufficient to explain tramadol-induced seizures alone. The inhibition of glutamate decarboxylase-mediated GABA synthesis has been reported to explain allylglycine-related seizuronic activity [52]. Here, the brain GABA concentrations and metabolism were not investigated as well as possible alterations induced by tramadol. This hypothesis appears unlikely to explain tramadol-related seizures, due to their rapid onset [16]. A recent experimental study suggested that preconditioning using ultra-low dose tramadol (2 mg/kg i.p.) protected rats against seizures following the administration 4 days later of 150 mg tramadol [53]. Protection was attributed at least in part to the up-regulation of neurochondrin, a neuron-specific cytosolic protein considered as a regulator of the metabotropic glutamate receptor-5 and to the down-regulation of the excitatory synaptic N-methyl-D-aspartate receptor subunit-1 and the subunit-1 of the postsynaptic α-amino-3-hydroxy-5-methyl-4-isoxazolepropionic acid receptor.

One of our study strengths was to investigate in vivo tramadol toxicity at elevated doses. Several limitations exist. The chosen doses of pretreatments were based on the literature data rather than on our own experiments and conditions, questioning whether the optimal doses of these agents were used. In this study, as usually recommended, six rats per group were used, thus presuming that any difference that would have required more rats to be established is not clinically pertinent. Regarding the brain monoamine study, sampling was limited to the peak time of seizures (~20–30 min post-tramadol injection), although it should be acknowledged that a series of time points correlated with the measured clinical parameters would have been preferable. Another issue consisted in the absence of measurement of seizing activity during PET acquisition and brain sampling for monoamine measurement.

It is also important to clarify the possible impacts of anesthesia with isoflurane on our PET findings. In the rat, anesthesia with isoflurane was shown to enhance the brain ^11^C-flumazenil binding [54]. The pons was used as reference region to estimate the *BP_ND_*. This approach is valid although the pons is a “pseudo-reference region” with limited specific ^11^C-flumazenil binding in the rat [28]. Here, potential changes in ^11^C-flumazenil binding induced by anesthesia with isoflurane were normalized to the ^11^C-flumazenil binding in the pons. Anesthesia with isoflurane is able to decrease monoamine release in the rat brain, including serotonin [55], histamine [56], dopamine [57] and norepinephrine [58]. The brain monoamine content during our PET investigation may have been impaired in comparison to the condition of non-anesthetized rat treated with tramadol.

## 4. Materials and Methods

### 4.1. Animals and Drugs

Male Sprague-Dawley rats (Janvier Labs, Le Genest-Saint-Isle, France) weighing 250–350 g at the time of experimentation were used, housed for 7 days before experimentation in an environment maintained at 21 ± 0.5 °C with controlled humidity and light-dark cycle. Food and tap water were provided ad libitum. Tramadol hydrochloride (Grünenthal, Aachen, Germany) and naloxone (Aguettant, Lyon, France) were diluted in sterile water to obtain solutions of 44 mg/mL and 0.4 mg/mL, respectively. Diazepam (Roche, Meylan, France) was diluted in 4% Tween (Tween-20^®^ diluted in 0.9% NaCl) to obtain a solution of 2 mg/mL. Cyproheptadine (Teofarma, Pavia, Italy) and fexofenadine (Sanofi-Aventis, Paris, France) were diluted in saline to obtain solutions of 5.3 and 15 mg/mL, respectively. ^11^C-flumazenil radiosynthesis and production for intravenous (i.v.) injection were performed as previously described [59,60] and diluted in saline to obtain 35.8 ± 4.9 MBq in a constant volume of 1 mL.

### 4.2. Jugular Catheterization

One week before the study, the rat jugular vein was catheterized using 60 cm-silastic tubing with external and internal diameters of 0.94 and 0.51 mm, respectively (Dow Corning Co., Midland, Michigan, USA), under ketamine (70 mg/kg) and xylazine (10 mg/kg) anesthesia. Catheters were tunneled subcutaneously and fixed at the back of the neck. Heparinized saline was injected into the catheter to avoid thrombosis and catheter obstruction. Rats were then returned to their individual cages for 7 days, allowing anesthesia washout and complete recovery. On the day of experiment and before drug administration, the catheter was exteriorized, purged, and its permeability checked.

### 4.3. Clinical Parameters

Temperature was measured using intraperitoneal (i.p.) implanted temperature transmitters (DSI, Amsterdam, The Netherlands). Sedation level based on a 4-stage scale from 0 (awake) to 3 (coma) was assessed [61]. At stage 0, rats were completely awake and their gait and righting reflexes were intact. At stage 1, rats had reduced activity, showed light impairment of gait and intact righting reflex with diminished muscle tonus. At stage 2, rats were asleep or static and showed reduced righting reflex. At stage 3, rats were comatose and did not have any righting reflex. Seizure severity was graded according to the modified Racine Score [62]. At stage 1, rats were immobile, with eyes closed, twitching of vibrissae and facial clonus. At stage 2, rats had head nodding associated with more severe facial clonus. At stage 3, rats had clonus of one forelimb. At stage 4, rats had rearing, often accompanied by bilateral forelimb clonus. At stage 5, rats had rearing with loss of balance and falling accompanied by generalized clonic seizures.

### 4.4. Measurement of Monoamine Concentrations in the Brain

Neurotransmitter concentrations were measured in the frontal cortex, the main area of serotoninergic projections that exhibited the prominent number of spike-wave discharges after 10 to 40 mg/kg tramadol administration [63]. Following tramadol administration, rats were immediately placed for 10 min in an anesthesia induction chamber (Tem Sega, Pessac, France) with 4% isoflurane (Virbac, Carros, France). They were exsanguinated to clean the brain of its blood and then decapitated. The frontal cortexes were collected 15–20 min after drug administration. Samples were frozen at −80 °C until measurement. Concentrations of monoamines, i.e., dopamine, serotonin, norepinephrine and their metabolites, 5-hydroxyindoleacetic acid (5-HIAA), 3-methoxy-4-hydroxyphenoglycol (MHPG), 3,4-dihydroxyphenylacetic acid (DOPAC) and homovanillic acid (HVA) were measured using high-performance liquid chromatography (HPLC) coupled to flurorimetry as previously described [64]. Radioenzymatic assay was used for histamine concentration determination as previously described [65].

### 4.5. ^11^C-Flumazenil Positron Emission Tomography (PET) Imaging

PET imaging acquisition was performed using an Inveon^®^ microPET-CT scanner (Siemens, Berlin, Germany) [66]. Rat anesthesia was induced and thereafter maintained using 3 and 1.5–2.5% isoflurane in O_2_, respectively, to insert a catheter in the caudal vein for ^11^C-flumazenil injection. Anesthetized rats were placed into the micro-PET-CT and a brain CT-scan was first performed. After CT completion, 90- or 60-min dynamic acquisitions were performed starting at the time of ^11^C-flumazenil intravenous (i.v.) injection, to study (i)—the impact of tramadol on the brain binding of ^11^C-flumazenil and (ii)—the mechanisms involved in tramadol-induced changes in ^11^C-flumazenil brain binding, respectively.

PET data were reconstructed using the FORE + OSEM2D algorithm, including normalization, attenuation, scatter and random corrections. PET images were co-registered using Pmod software (version 3.6, Zürich, Switzerland) to a brain magnetic resonance imaging (MRI) template published by Schiffer et al. [67] (normalization of PET-CT on MRI and application of the transformation to the PET images). This template incorporates adult male Sprague-Dawley rats (250–300 g) and implements the Paxinos coordinates. Time activity curves (TACs) were generated in different brain regions predefined in the Schiffer’s template. Radioactivity concentration was expressed as the percentage of the injected dose (%ID.mL^−3^) versus time (min). The binding potential (*BP_ND_*) of ^11^C-flumazenil in selected brain regions (*BP_ND_* = B_max_/K_D_, where B_max_ represents the total density of receptors in the tissue and K_D_, the radioligand equilibrium dissociation constant) was estimated using the simplified reference tissue model (SRTM) and the pons as the reference region [68]. Corresponding parametric PET images were generated using SRTM to visualize ^11^C-flumazenil binding (*BP_ND_*) to GABA_A_ receptor (PXMOD, Pmod Software).

### 4.6. Study Design

#### 4.6.1. Study 1-Effects of Pretreatments on Tramadol-Induced Sedation, Seizures, Temperature and Brain Monoamines

Catheterized rats were randomly assigned to five groups to receive various agonists or antagonists (Table 1), before i.p. 75 mg/kg tramadol, a dose able to induce typical poisoning features [16].

Numbers were assigned to each pretreatment group chosen randomly for each rat. Investigators were not blinded to the administered treatment. Pharmacological doses of the different pretreatments were selected in agreement with previous publications: i.p. 1.77 mg/kg diazepam, a positive allosteric modulator of the GABA_A_ receptor at the benzodiazepine binding site [69,70]; i.v. 2 mg/kg bolus followed by 4 mg/kg/h infusion naloxone, a mu-, delta- and kappa-opioid receptor antagonist [71]; i.p. 10 mg/kg cyproheptadine, a serotonin-2A and -2C receptor antagonist [70,72]; and i.p. 15 mg/kg fexofenadine, a histamine-1 receptor antagonist [73]. Tramadol was injected after each pretreatment with a delay chosen to allow for its peak effects. In the first series, the clinical parameters described above were measured in the five rat groups (N = 6/group) immediately before tramadol injection and at 5, 10, 15, 30, 45, 60, 90 and 120 min after injection. In a second series, brain monoamine concentrations were measured at 15–20 min after tramadol administration, when seizures were the most frequent. A sixth group serving as a control group with rats receiving saline i.p. instead of tramadol was considered for the monoamine assays.

#### 4.6.2. Study 2-Interactions of Tramadol with GABA_A_ Receptors

Interaction of tramadol with the GABAergic system was assessed using PET imaging and ^11^C-flumazenil. Rats were randomly assigned to two groups (N = 5/group) to receive 75 mg/kg i.p. tramadol (Tramadol group) or 1.7 mL of sterile water i.p. (Vehicle group), 15 min before ^11^C-flumazenil injection followed by 60 min PET acquisition.

Displacement experiments were performed to address the direct competition of tramadol with ^11^C-flumazenil on its binding site [35]. To that end, rats were randomized into four groups (N = 2/group) to receive i.v. ^11^C-flumazenil alone (Baseline group) and in addition, 1 mg/kg tramadol (Tramadol-1 group), 25 mg/kg tramadol (Tramadol-25 group) or 1 mg/kg diazepam (Diazepam group), injected i.v. during PET acquisition 30 min after ^11^C-flumazenil injection.

### 4.7. Statistical Analysis

The results are expressed as median and quartiles (Study 1) and mean ± SEM (Study 2). To permit the simultaneous analysis of the effects of time and treatments on sedation, temperature and monoamine concentrations (Study 1), the area under the curve (AUC) from T0 to the completion of measurement (120 min) was calculated for each animal and each studied parameter, using the trapezoid method. Thereafter, for sedation and temperature, the AUCs were compared using Kruskal-Wallis tests for comparisons between five groups. For monoamine concentrations, we compared the AUCs using Mann–Whitney U-tests for comparisons two-by-two. Regarding the effects of treatments on seizures, comparisons were performed using two-way analysis of variance followed by multiple comparison tests using Bonferroni’s correction. In the PET-imaging study, the *BP_ND_* in the two studied groups were compared using Mann–Whitney U-tests. All tests were performed using Prism version 6.0 (GraphPad Software, San Diego, CA, USA). *p*-values < 0.05 were considered as significant.

## 5. Conclusions

The present study demonstrated that tramadol-induced seizures are only prevented by diazepam, a positive allosteric modulator of GABA_A_ receptor. The serotoninergic, histaminergic, opioidergic, dopaminergic, and norepinephrinergic pathways seem unlikely to be involved. Our data highly suggest that tramadol-induced seizures result from tramadol’s interaction with the GABA_A_ receptor involving a noncompetitive mechanism at the benzodiazepine-binding site. Our findings suggest that a strategy primarily relying on benzodiazepines may be appropriate in the management of tramadol-induced seizures.

## Figures and Tables

**Table 1:** Treatments administered in the different study groups.

	Group	Saline + Tramadol Group	Diazepam + Tramadol Group	Naloxone + Tramadol Group	Cyproheptadine + Tramadol Group	Fexofenadine + Tramadol Group	Control Group
*Time (min)*							
T-30		ø	ø	ø	ø	15 mg/kg fexofenadine i.p.	ø
T-15		4% tween i.p.	1.77 mg/kg diazepam i.p.	4% tween i.p.	10 mg/kg cyproheptadine i.p.	ø	4% tween i.p.
T-10		Saline i.v.	Saline i.v.	2 mg/kg i.v. bolus followed by 4 mg/kg/h infusion	Saline i.v.	Saline i.v.	Saline i.v.
T0		75 mg/kg tramadol i.p.	75 mg/kg tramadol i.p.	75 mg/kg tramadol i.p.	75 mg/kg tramadol i.p.	75 mg/kg tramadol i.p.	Saline i.p.

i.p., intraperitoneal; i.v., intravenous.

## Data Availability

Data is contained within the article.
